# Reproducibility and day time bias correction of optoelectronic leg volumetry: a prospective cohort study

**DOI:** 10.1186/1471-2288-11-138

**Published:** 2011-10-05

**Authors:** Rolf P Engelberger, Claudia Blazek, Felix Amsler, Hong H Keo, Frédéric Baumann, Werner Blättler, Iris Baumgartner, Torsten Willenberg

**Affiliations:** 1 Swiss Cardiovascular Center, Division of Clinical and Interventional Angiology Inselspital, University Hospital and University of Bern, Switzerland; 2 Amsler Consulting, Basel, Switzerland; 3 Academic Section of Vascular Surgery, Imperial College School of Medicine, Charing Cross Hospital, London, UK

## Abstract

**Background:**

Leg edema is a common manifestation of various underlying pathologies. Reliable measurement tools are required to quantify edema and monitor therapeutic interventions. Aim of the present work was to investigate the reproducibility of optoelectronic leg volumetry over 3 weeks' time period and to eliminate daytime related within-individual variability.

**Methods:**

Optoelectronic leg volumetry was performed in 63 hairdressers (mean age 45 ± 16 years, 85.7% female) in standing position twice within a minute for each leg and repeated after 3 weeks. Both lower leg (leg_BD_) and whole limb (limb_BF_) volumetry were analysed. Reproducibility was expressed as analytical and within-individual coefficients of variance (CV_A_, CV_W_), and as intra-class correlation coefficients (ICC).

**Results:**

A total of 492 leg volume measurements were analysed. Both leg_BD _and limb_BF _volumetry were highly reproducible with CV_A _of 0.5% and 0.7%, respectively. Within-individual reproducibility of leg_BD _and limb_BF _volumetry over a three weeks' period was high (CV_W _1.3% for both; ICC 0.99 for both). At both visits, the second measurement revealed a significantly higher volume compared to the first measurement with a mean increase of 7.3 ml ± 14.1 (0.33% ± 0.58%) for leg_BD _and 30.1 ml ± 48.5 ml (0.52% ± 0.79%) for limb_BF _volume. A significant linear correlation between absolute and relative leg volume differences and the difference of exact day time of measurement between the two study visits was found (P < .001). A therefore determined time-correction formula permitted further improvement of CV_W_.

**Conclusions:**

Leg volume changes can be reliably assessed by optoelectronic leg volumetry at a single time point and over a 3 weeks' time period. However, volumetry results are biased by orthostatic and daytime-related volume changes. The bias for day-time related volume changes can be minimized by a time-correction formula.

## Background

Lower extremity edema are a cardinal symptom of chronic venous insufficiency (CVI) but may also appear under various other conditions [1,2]. A reliable and easy to use tool to quantify the severity of peripheral edema is needed for monitoring disease evolution or effects of therapeutic interventions [3]. In practice, leg circumference measurements are performed with a tape measure [4]. One of these tapes, the leg-O-meter has been validated showing reproducible results [5]. Nevertheless, it allows measurement at a single level of the leg only and may not give information about the true leg volume. Therefore, more sophisticated methods for leg volumetry have been introduced. There are straightforward volume measurements available which are based on the frustrum method (i.e. modelling the leg as a section of a cone and applying a geometric formula), rapid, but not very accurate [4]. Water displacement volumetry is a highly reproducible method and is regarded as the gold standard [6-8]. However, its use is cumbersome and errors of execution may falsify the results [7]. New optoelectronic devices have been developed. They are sophisticated and rather expensive but very easy to use. One of these devices is the Perometer^® ^(Pero-System Messgeräte GmbH, Wuppertal Germany) which is able to measure leg volumes in standing and supine position noninvasively and repeatedly within short time intervals, each measurement taking less than a minute [9-11]. Several studies have compared the Perometer^® ^device with the water displacement volumetry in different clinical situations and underlined the validation of this mode of volumetry [12-14]. To date, however, repeatability of optoelectronic leg volume measurements at single and at different times has not been studied appropriately according to current standards [15-18]. The latter poses a critical issue as leg volume tends to increase during the day [3,19]. This physiologic and, when more accentuated, pathologic phenomenon affects comparison of leg volume values at different days when the exact time of assessment is not taken into consideration. This day-time dependence of leg volume assessment may limit the use of the measuring device and complicate study protocols when pharmaceutical or physical therapies are investigated for their ability to reduce leg edema over a certain time period.

The primary aim of this study was to investigate the reproducibility of leg volume measurements by the Perometer^® ^device at a single time point and after a 3 weeks' time period in a large cohort of healthy individuals. Secondly, we present a formula which allows elimination of day-time related within-individual variability of optoelectronic leg volumetry.

## Methods

The study was approved by the ethics committee of the University of Bern and conformed with the principles outlined in the Declaration of Helsinki. All participants granted written informed consent.

### Study population

The study was set up as a single centre, prospective cohort study with individuals at occupational risk for developing leg symptoms [20]. Therefore, hairdressers working in the Bern area, older than 18 years of age, were eligible. Exclusion criteria were inability to stand, personal history of deep vein thrombosis or peripheral arterial disease, pregnancy and unwillingness or inability to give written informed consent. No particular instructions for break time, working hours or physical activity were given to the participants. Recruitment was performed by contacting all members of the regional hairdressers association by mail.

### Measurement of leg volume

For leg volume assessment an optoelectronic scanner was applied (Perometer^®^, Pero-System Messgeräte GmbH, Wuppertal Germany). The instrument consists of a movable measurement frame that is mounted on a linear guide and connected to a computer which runs the associated software. The sensors are built in the frame which is moved over the length of the leg of an up-right person. The frame carries light emitters each facing a corresponding detector. Light switches are mounted perpendicular to each other. The arrays are interrupted by the limb during movement of the frame without any skin contact, and every 4.7 mm a new measurement is performed. Silhouette images are taken from the front and side and an elliptic cross-sectional area is calculated which forms the base of a 4.7 mm thick slice. The circumference of each slice is plotted against the distance from the sole of the foot establishing a profile of circumference and volume. The leg volume between two given points is integrated from the volumes of all slices between these points. By analyzing the leg profile, the software automatically indicates some anatomic landmarks, such as the smallest circumference at the ankle level (B-measure), the largest circumference of the lower leg (C-measure), the calf circumference right below the knee bend (D-measure) or the mid-thigh circumference (F-measure). Two leg volumes were assessed in our study: the lower leg without the foot, from B-measure to D-measure (leg_BD_) and the limb volume without the foot up to the mid-thigh region, hence from the B-measure to the F-measure (limb_BF_).

### Study protocol

Leg volumetry was performed at two different study days scheduled 3 weeks apart. For their convenience participants were allowed to decide themselves at what time between 8 am and 6 pm they wanted to conduct each of their study visit. All participants were examined by a vascular medicine specialist and underwent leg volume measurement using the Perometer^®^. Volumetry was performed in standing position with the body weight equally distributed on either leg, after being sitting relaxed on a chair for a standardized time period of 15 minutes. Shoes and socks were removed before the sitting period [7]. All measurements were conducted by the same research fellow (CB) in a room with constant temperature between 23 - 25°C. Duplicate measurements were taken immediately one after the other for the left and the right leg. At visit 2 (V2) leg volume measurements were repeated according to the same procedure described above.

### Statistical analysis

The distribution of baseline demographic data and clinical characteristics are presented as the mean (+/- SD) or as percentages. Following the concepts described by Bland and Altman [15,16,21], as well as Fraser and Harris [17], we computed estimates of the analytical variance (*s*_*A*_^*2*^), the average variance of repeated measurements at the same time point, also known as measurement error [15] and the within-individual variance (*s*_*W*_^*2*^), the average variance of repeated measurement in the same subject at different time points by performing a one-way analysis of variance (ANOVA). The different variances were transformed into the corresponding coefficient of variances (CV_A_, CV_w_) by calculating the square root of the respective variance estimate, divided by the overall mean, and expressed as percentage. The intra-class correlation coefficient (ICC) was calculated as an overall estimation of the reproducibility of leg volume measurements [16,22]. Linear regression analysis was performed to assess the relationship between leg volume change and the exact daytime of leg volume measurement at V1 and V2. For this calculation, all measurements of the right and the left leg were pooled. Subsequently, the leg volume measurements of visit 2 were adjusted with the time-correction formula created according to the linear regression analysis. Within-individual coefficient of variance was recalculated with the corrected values. Paired t-tests were performed to compare the mean absolute and percentage volume change between V1 and V2 with and without correction of the values with the time-correction formula. All statistical analyses were performed using STATA version 9.1 (StataCorp, College Station, Texas, USA). P-values of < .05 were considered statistically significant.

## Results

### Study population

The study was performed between September 2009 and March 2010. Sixty-three participants were included and the baseline characteristics of these participants are outlined in Table 1.

**Table 1:** Demographic and clinical characteristics of the 63 study participants

Age (years)	45	±	16
Females	54		(85.7%)
BMI (kg/m2)	22.9	±	3.5
			
Occupation* (%)	82.9	±	24.9
Working hours spent upright (%)	88.0	±	11.3
Regular physical exercise (%)	66.7	±	47.5
			
Severity of venous disease (CEAP classification)			
C0 (No objective findings)	5		(7.9%)
C1 (Teleangiectasies only)	47		(74.6%)
C2 (Varicose veins only)	6		(9.5%)
C3 (edema with or without varicose veins)	5		(7.9%)

Data are means ± SD or number (%); BMI, body mass index; * Occupation of 100% corresponds to 42 working hours/week

### Leg volume measurements

At each study visit, the volume of each leg was measured twice within half a minute. A total of 492 leg volume measurements were performed. The averaged volumetric data obtained at the two visits are summarized in Table 2. Comparison of results obtained at V1 and V2, revealed a non-significant decrease of 5.9 ml ± 44.3 ml (P = .14) in leg_BD _while the limb_BF _showed a non-significant increase of 10.9 ml ± 113.6 ml (P = .15).

**Table 2:** Leg volume measurements, derived coefficients of variation and intra-class correlation coefficients, before and after correction with time-volume formula

		leg volume measurement		Crude values			after correction with time-volume formula	
		
		visit 1	visit 2	**CV**_**a **_**[%]**	**CV**_**w **_**[%]**	ICC	**CV**_**w **_**[%]**	ICC
limb_BD_	left leg	2295.7 ± 407.7	2283.7 ± 399.8*	0.41	1.3532	0.9941	1.1956	0.9954
	right leg	2298.0 ± 415.8	2298.1 ± 402.8	0.55	1.3848	0.9939	1.3211	0.9954
leg_BF_	left leg	5841.8 ± 821.4	5841.0 ± 811.5	0.68	1.3222	0.9920	1.2056	0.9933
	right leg	5896.2 ± 807.9	5919.0 ± 807.6	0.68	1.4005	0.9904	1.3634	0.9909

CV coefficient of variance, a analytical, w within-individual, ICC intra-class correlation coefficient, limb_BD _lower limb without the foot (from B-measure to D-measure), leg_BF _leg volume without the foot (from the B-measure to the F-measure). leg volumes as means ± SD; * *p *< 0.05 versus visit 1

The comparison of the two leg volume measurement at the first visit yielded significantly higher values at the second measurement for both leg_BD _and limb_BF _(P *<*.01 for all pairs). The mean absolute and relative volume increases between the first and the second leg volumetry are summarized in Table 3. No significant difference in absolute or relative volume increase between the right and the left side for both leg_BD _and limb_BF _were observed (P = .08 for leg_BD _and P = .85 for limb_BF_). Compared to the mean absolute and relative increase between the first and the second leg_BD _measurement, the corresponding limb_BF _increases were significantly higher (P *<*.001 for both). At V2, similar absolute and relative increases between the first and second leg volume measurements were found for both leg_BD _and limb_BF _(data not shown).

**Table 3:** Comparison of the two leg volume measurements at visit 1*

		volume difference between the 1^st ^and 2^nd ^measurement	
		
		absolute [ml]	relative [%]
leg_BD_	left leg	+ 5.2 ± 12.4	+ 0.24 ± 0.52
	right leg	+ 9.5 ± 15.3	+ 0.42 ± 0.63
	***mean of both legs***	**+ 7.3 ± 14.1**	**+ 0.33 ± 0.58**
limb_BF_	left leg	+ 29.2 ± 48.8	+ 0.52 ± 0.80
	right leg	+ 31.0 ± 48.7	+ 0.51 ± 0.79
	***mean of both legs***	**+ 30.1 ± 48.5**	**+ 0.52 ± 0.79**

* Leg volumetry was performed twice within 30 seconds in standing position after being sitting relaxed on a chair for a standardized time period of 15 minutes. leg_BD _lower limb without the foot (from B-measure to D-measure), limb_BF _leg volume without the foot (from the B-measure to the F-measure). Values as means ± SD

### Relation between day time of measurement and leg volume

**Figure 1:**
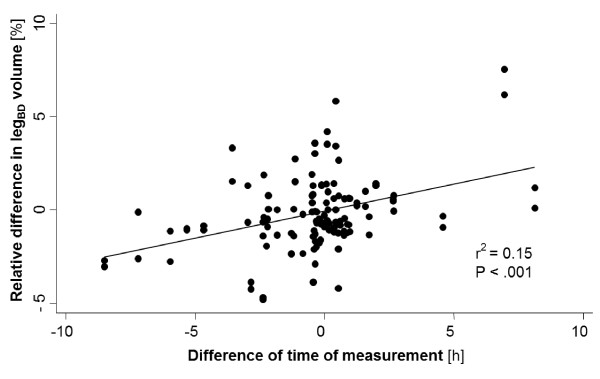
**Relative leg**_**BD **_**difference between visit 1 and 2 (calculated by: [leg**_**BD **_**visit 2 - leg**_**BD **_**visit 1]/leg**_**BD **_**visit1) plotted against the time difference of measurement (calculated by: exact daytime of measurement at visit 2 - the exact daytime of measurmeent at visit 1) **. leg_BD_, lower leg without the foot (from B-measure to D-measure)

**Figure 2:**
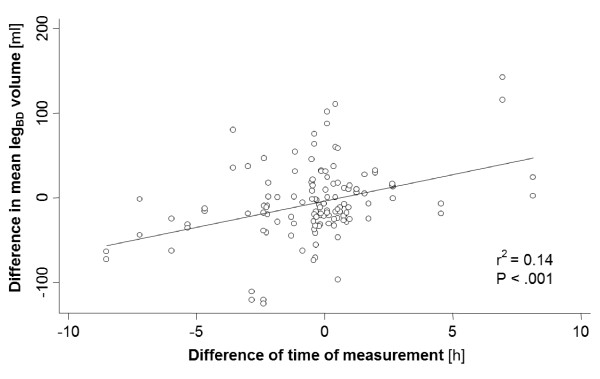
**Absolute leg**_**BD **_**difference between visit 1 and 2 (calculated by subtracting the leg**_**BD **_**from visit 2 minus leg**_**BD **_**from visit 1) plotted against the time difference of measurement (calculated by: exact daytime of measurement at visit 2 - the exact daytime of measurmeent at visit 1) **. leg_BD_, lower leg without the foot (from B-measure to D-measure)

We found a significant linear correlation between the exact daytime and leg volume measurements for both leg_BD _(r^2 ^= 0.05, P = .01) and limb_BF _(r^2 ^= 0.08, P = .001). After statistical adjustment for body mass index (BMI), this correlation was no longer significant. Furthermore, a significant linear correlation was found for the leg volume change (absolute and relative) between the two visits and the difference of exact time of leg volume measurement between the two visits, as illustrated in Figure 1 and 2. Adjustment for BMI, age, sex, and severity of venous disease according to the CEAP classification did not alter this correlation. For each hour of difference between the exact measurement time points at each visit, a mean absolute leg_BD _difference of 6.2 ml (95% CI: 3.5 - 9.0 ml) was observed (r^2 ^= 0.14, P < .001), corresponding to a mean relative leg_BD _difference of 0.29% (95% CI: 0.16 - 0.41%, r^2 ^= 0.15, P < .001) per hour of difference. This resulted in a time-correction formula for the leg_BD_:

$$\Delta_{\%\text{corr}}=\Delta_{\%\text{meas}}-\left(0.\text{288}*\Delta_{\text{t}}\right),$$

where Δ_% meas _is the observed percentage of volume difference between the visits (calculated by: [leg volume at V2 - leg volume at V1]/leg volume at V1), and Δ_t _the absolute difference in hours of the time point of measurement at the different visits (calculated by: exact time at V2 - exact time at V1) after transformation of daytime from hours:minutes into a decimal number. The following example with a new "edema-limiting drug" will illustrate this formula: at baseline V1, leg_BD _3000 ml, measurement time at 9:30 h (corresponding to 9.50 h in decimal system); after 3 weeks of treatment, follow-up at V2: leg_BD _3020 ml, measurement time at 14:45 h (= 14.75 h in decimal system) → Δ_%meas _= (3020 ml - 3000 ml)/3000 ml = +0.67% and Δ_t _= 14.75 h - 9.50 h = 5.25 h → Δ_%corr _= +0.67% - 0.288 * 5.25 = -0.85%. Interpretation of the example: the observed volume increase between V1 and V2 of +0.67% is misleading and referable to the later measurement time at V2. After correction of the expected daytime volume increase, the drug in fact may provide some effect with a corrected volume change of -0.85%.

For limb_BF _measurements, the mean absolute volume difference for each hour of difference between the exact measurement time points was 14.6 ml (95% CI: 7.4 - 21.8 ml), and in relative terms 0.26% (95% CI: 0.14 - 0.38%). The corresponding formula for the limb_BF _is therefore:

$$\Delta_{\%\text{corr}}=\Delta_{\%\text{meas}}-\left(0.\text{ 259}*\Delta_{\text{t}}\right).$$

### Analytic and within-individual coefficient of variance

The analytic coefficients of variance for both leg_BD _and limb_BF _measurements appeared to be low for both sides with a slightly higher CV_A_ for the whole limb volumetry as shown in Table 2. Within-individual coefficients of variance were less than 2% on each side for leg_BD _and limb_BF _measurements (Table 2). Intra-class correlation coefficients for both leg_BD _and limb_BF _measurements drew near 1.0 for either side (Table 2).

After adjustment of the leg volume measurements at V2 with the above defined time-correction formula, within-individual coefficient of variance for both legs appeared to be even lower than without adjustment (Table 2).

The calculated intra-class correlation coefficients rose slightly with the adjusted values (Table 2). The mean difference between V1 and V2 of mean absolute leg_BD _measurements was significantly smaller with the time-corrected values than without time correction for both leg_BD _in absolute (-3.3 ml versus -5.9 ml, P = .045) and relative terms (-0.13% vs -0.33%, P = .045) while no such difference was observed for limb_BF _measurements (P = .09).

## Discussion

In the present work we report the leg volume assessment with an optoelectronic device (Perometer^®^) in a large cohort of hairdressers. Our results suggest that leg volumetry with the Perometer^® ^device is highly reproducible, not only at a single time point but also if measurement is repeated 3 weeks later. Moreover, we observed a strong correlation between the leg volume and the exact day time of measurement. By applying a here presented time-correction-formula, this time-dependency can be eliminated with a further increase of reproducibility. Our findings are of practical interest because they indicate that changes of leg volume over time can reliably be monitored with an easy to use optoelectronic Perometer^® ^device. Daytime-correction enables longitudinal leg volume assessments by simplifying the complicating relationship between leg volume and day time.

Leg edema is a manifestation of a broad spectrum of pathologies. Since leg edema is usually reversible, at least in its early stages, serial leg volume measurements can be used to assess the therapeutic effect of various interventions. However, expected leg volume changes over time in clinical studies are rather low [7] and therefore demand a reliable measurement tool [3,23,24]. For instance, in a recently published study by Rabe and collaborators evaluating the effect of a red-vine-leaf extract on changes in lower leg volume assessed by water displacement volumetry, a mean absolute decrease of 27 ml (corresponding to a relative decrease of 1.18%) in the treatment group and 7.2 ml (0.31%) in the placebo group after 12 weeks of treatment was observed [23]. Water displacement volumetry is considered as the gold standard despite some important limitations since it is rather time consuming, subject to different error sources [7] and cannot be applied to patients with skin ulcers, a common complication of advanced CVI [4]. Optoelectronic systems such as the Perometer^® ^device used in the present study are easy and reliable as an alternative method and have been validated against water displacement volumetry [9,13,14].

### Analytic and within-individual variance

Our results demonstrate that the analytic coefficient of variance, or expressed differently, the measurement error is minor with a value around 0.5% for lower leg and around 0.7% for whole limb volumetry. Similar results were found with the water-displacement volumetry with coefficients of variations ranging from 0.32-1.56% [3]. Moreover, the within-individual variance seen in our analyses is acceptable with a value of around 1.4% for both lower leg and whole limb volumetry. This becomes more obvious when considering that some in daily practice commonly measured biochemical markers such as total cholesterol show much higher analytic and within-individual coefficients of variance (CV_A _1% and CV_w _8%) [25].

Another possibility to estimate the reproducibility of repeated measurement is the calculation of the intra-class correlation coefficient (ICC) [26]. Our results show excellent ICC's for both lower leg and whole limb volumetry with values close to 1, the maximum value of ICC that signifies little variation within the subjects compared to the variation between the subjects [26].

### Day time bias correction

Several previous studies showed that leg volume tends to increase during daytime, even in healthy subjects [3,19]. In line with these observations, we found a linear correlation between the exact daytime of measurement and the leg volume in our study. This correlation was no longer significant after adjustment for BMI. However, many studies defining leg volume as an endpoint report absolute or relative leg volume differences between different measurement time points [3,23,24,27]. Our data indicate that variation of the exact daytime of volumetry measurements at the different visits features a highly significant linear correlation between the time difference and the leg volume difference, even after adjustment for possible confounders such as the BMI. This linear correlation allows the creation of a simple time-correction formula, which enables arithmetical correction of day- time dependency. Although the correction coefficients seem to be relatively small, time-correction is still important because of the rather small volume modifications observed in clinical studies [7]. After day-time correction of leg volume measurements performed at the second visit with application of the presented formula, the within-individual coefficient of variance for both lower leg and whole limb volumetry showed further improvement. The consequence of these findings is not only the increase of reproducibility over time, but also a simplification for the monitoring of leg volume changes over a certain time period by eliminating the day-time of measurement as a confounding factor.

The relatively low coefficient of determination (r^2^) for the correlation between daytime of volumetry measurement and the leg volume suggests that other factors not assessed in our study (e.g. physical activity or working hours before measurement) or still unknown factors seem to have an important influence on the observed volume changes over time. However, the exact daytime of volume measurement has the inherent advantage that it can very easily be recorded without any risk of subjectivity.

### Immediate volume increase in standing position

A further finding of our analysis is the immediate leg volume increase when measured twice within 30 seconds in a standing position after a standardized 15-minute period of relaxed sitting. This could potentially represent a lack of reliability; however, we strongly suggest a rapid orthostatic leg volume change to explain this observation. Several previous studies observed a rapid increase of leg volume when body position is changed from a supine to a sitting or standing position [10,27-29], with no difference between healthy and varicose legs [10,28]. After changing position from lying to sitting/standing, Stick et al. reported a two-stage change in calf volume with a fast initial increase followed by a slow continuous volume increase [30]. The mean calculated rates of this slow increase were 0.17% per minute during standing and 0.12% per minute during sitting, respectively. Although the two measurements in our study were performed within less than one minute, an even higher percentage of volume increase between the first and the second measurement was observed. This might be explained by our time point of volume measurements immediately after changing position from sitting to standing and corresponds to the rapid volume increase period as reported by Stick et al. While most other studies assessed the orthostatic leg volume increase after a change from lying to standing position, in our study volumetry was performed immediately after a change from sitting to standing. A similar protocol was adapted by Pannier et al., however, in the latter study the participants were sitting relaxed for 15 minutes with raised legs [10]. Therefore it can be assumed that the baseline venous filling between raised legs in the study by Pannier and our study may differ, not allowing a direct comparison. As a practical general consequence of all these different studies, it appears to be crucial to apply a standardized measurement protocol, with particular attention to position changes and timing when leg volumetry is used in clinical practise or in a research setting.

### Limitations

Our study has several limitations. First, the study population included relatively young subjects working mainly in standing position, most of them without any clinical evidence of superficial venous reflux. Therefore, extrapolations from our findings to other clinical settings may be critical. However, Pannier et al. did not observe any difference between varicose legs and legs without varicosity [10], using the same Perometer^® ^device for orthostatic volume change measurements. Second, all participants were hairdressers, a population which might be considered to have an elevated risk for leg symptoms because of a mostly standing working position [20]. Interestingly, compared to the recent Bonn Vein Study investigating the prevalence of CVI in 3072 urban and rural residents in Germany, our hairdresser group showed a slightly lower prevalence of varicose veins (CEAP C2, 9.5% versus 14.3% in the Bonn Vein Study) and leg edema (CEAP C3, 7.9% versus 13.4%) while telangiectasies were more frequent in the hairdresser group (CEAP C1, 74.6% versus 59%). Third, all leg volume measurements were performed by the same investigator (CB). It cannot be excluded that reproducibility would be lower when another source of variability, namely interobserver variability, would be added. However, since the utilization of the Perometer^® ^device is very easy and straight forward with an automatically calculated leg volume by a software program, interobserver variability is likely to be remote.

## Conclusions

In conclusion, our findings show that intra-observer reliability of leg volume measurements with the easy to apply optoelectronic Perometer^® ^device is very high, not only at a single time point but also over a three weeks period. Furthermore, observed leg volume changes over time can be confounded by the daytime of volume measurement. With the presented time-correction formula, this confounding factor can be minimized. In addition to the daytime dependency, another source of variability is orthostatic leg volume change which is best avoided by applying the same strictly standardized measurement protocol for all leg volume assessments and by taking the mean of at least two measurements at each visit.

## List of Abbreviations used

BMI: Body Mass Index; CEAP: Clinical Etiological Anatomical Pathophysiological; CV: coefficients of variance; CVI: chronic venous insufficiency; ICC: intra-class correlation coefficients; SD: standard deviation; V: visit

## Competing interests

The authors declare that they have no competing interests.

## Authors' contributions

RPE participated in the analysis and interpretation of the data, and drafted the manuscript. CB participated in the study design and data collection. FA participated in the study design and the statistical analysis. HHK participated in the data analysis and data interpretation. FB participated in the data analysis. WB conceived the study and helped to draft the manuscript. IB participated in the study design and data analysis. TW conceived the study, participated in the data analysis and hold the overall responsibility. All authors read and approved the final manuscript.

## Pre-publication history

The pre-publication history for this paper can be accessed here:

http://www.biomedcentral.com/1471-2288/11/138/prepub
